# From Biofortification to HT-29 Growth Inhibition: Selenium-Enriched Sunflower Sprouts Modulate Apoptosis- and Cell Cycle-Related Markers

**DOI:** 10.3390/foods15142539

**Published:** 2026-07-17

**Authors:** Piyanete Chantiratikul, Anut Chantiratikul, Yada Laotongsan, Gondanai Lasungneon, Worachot Saengha, Thipphiya Karirat, Piyathida Promjamorn, Nyuk Ling Ma, Vijitra Luang-In

**Affiliations:** 1 Department of Chemistry, Faculty of Science, Mahasarakham University, Kantarawichai District, Maha Sarakham 44150, Thailand; piyanete.c@msu.ac.th (P.C.); jbjew2542@gmail.com (Y.L.); 2 Department of Agricultural Technology, Faculty of Technology, Mahasarakham University, Kantharawichai District, Maha Sarakham 44150, Thailand; anut.c@msu.ac.th; 3 Natural Antioxidant Innovation Research Unit, Department of Biotechnology, Faculty of Technology, Mahasarakham University, Kantarawichai District, Maha Sarakham 44150, Thailand; gondanai.l@msu.ac.th (G.L.); worachot207@gmail.com (W.S.); thipphiya.k@gmail.com (T.K.); piyathida.pprom@gmail.com (P.P.); 4 BIOSES Research Interest Group, Faculty of Science and Marine Environment, Universiti Malaysia Terengganu, Kuala Nerus 21030, Terengganu, Malaysia; nyukling@umt.edu.my

**Keywords:** colorectal cancer cell line, cytotoxicity, *Helianthus annuus* L., metabolomics, selenium biofortification, wound closure inhibition

## Abstract

Selenium biofortification of edible sprouts is a promising strategy to enhance the cancer cell growth-inhibitory potential of plant-based foods. This study investigated the effects of selenium (Se)-enriched sunflower sprout extracts on human colorectal adenocarcinoma HT-29 cells in comparison with non-enriched sprouts. Cytotoxicity was evaluated by MTT assay, clonogenic survival by colony formation assay, wound closure by wound-healing assay, apoptosis- and cell cycle-related gene expression by qRT-PCR, and protein expression by Western blotting. Metabolite profiles were characterized using LC-MS/MS-based untargeted metabolomics. Se enrichment markedly enhanced cytotoxicity, reducing the IC_50_ from 413.80 ± 6.00 (control) to 152.90 ± 6.00 µg/mL and increasing Emax from 59.29 ± 0.11% (control) to 75.70 ± 0.49%. The Se-enriched extract showed greater inhibition of colony formation (IC_50_ 53.72 ± 1.12 µg/mL) and wound closure (IC_50_ 76.07 ± 0.06 µg/mL). At the molecular level, Se-enriched sprouts upregulated *Bax*, *caspase-3* and *p21*, downregulated *Bcl-2*, *CDK4*, *NF-κB p65* and *MMP-9*, and increased Bax and p21 protein levels, linked with apoptosis-related signaling and cell cycle modulation. Metabolomics revealed significant increases in purine-related metabolites, lysophosphatidylcholine (LPC) 18:3, and choline, alongside decreases in several quinic acid derivatives and related phenolic metabolites. Se enrichment enhanced the in vitro antiproliferative activity of sunflower sprout extract in HT-29 cells and may modulate apoptosis- and cell cycle-related markers. These findings support further mechanistic and in vivo investigations of Se-enriched sunflower sprouts as a candidate functional food ingredient.

## 1. Introduction

Colorectal cancer (CRC) is among the leading causes of cancer mortality worldwide, and chemoprevention through diet remains an important complementary strategy to screening and therapy [1,2,3]. Higher intake of dietary antioxidants, including selenium (Se), has been associated with a reduced incidence of CRC and improved survival in patients, although the strength of evidence varies and depends on the dose and source [1,3,4,5]. Functional plant foods that combine Se with phytochemicals are therefore of particular interest for CRC prevention.

Sprouts and microgreens are established candidate functional foods with high micronutrient and polyphenol content, and represent a well-validated matrix for Se biofortification [6,7,8,9,10,11]. Se-biofortified microgreens of kale, kohlrabi, coriander, basil, tatsoi, pea and alfalfa can reach 28–133 µg Se g^−1^ DW and cover 20–133% of the human Se recommended daily allowance, while also increasing total phenolics by up to 95% and enhancing antioxidant capacity without severe growth penalties at optimized doses [6,7,8,9,10,11]. In several species, Se enrichment stimulates phenylpropanoid metabolism, increasing compounds such as chlorogenic acid, caffeic acid, rutin and kaempferol glycosides that are relevant to cancer chemoprevention [7,9,11].

Beyond nutritional enrichment, Se-containing forms—whether as organic Se in plants or as Se nanoparticles—exert direct in vitro cancer cell inhibitory effects in colon cancer models. Biogenic or polysaccharide stabilized Se nanoparticles inhibit HT-29 cell viability with IC_50_ values down to 19–20 µg/mL, induce 20–30% apoptosis, elevate *Bax* and *caspase-3/9*, reduce *Bcl-2*, and cause cell cycle arrest and loss of mitochondrial membrane potential [12,13,14,15]. These effects are accompanied by increased reactive oxygen species and, in some cases, induction of immunogenic cell death markers [13,15]. Such data indicate that appropriately delivered Se can selectively impair colon cancer cells while sparing normal cells [13,14,15].

Recent work in Se biofortified sprouts (kale, kohlrabi, pea, alfalfa) has focused primarily on agronomic performance, Se speciation, antioxidant status and general health value [6,7,8,9,10,11]. Sunflower sprouts have previously demonstrated favourable phytochemical profiles for health benefits [16,17]. Thus, Se-enriched sunflower sprouts would improve their nutritive value. The previous report revealed that the optimal selenate concentration for Se-enriched sunflower sprout production was 2.0 mg/L [16]. However, much less is known about whether Se-enriched sunflower sprouts can inhibit in vitro cancer cell growth, migration and clonogenic survival in human CRC cells, and which metabolites are modulated.

Accordingly, this study aimed to evaluate the in vitro cancer cell growth-inhibitory activity of Se-enriched sunflower sprout extracts against HT-29 human colorectal cancer cells in comparison with non-enriched sunflower sprout extracts. Specifically, the study investigated their effects on cell cytotoxicity, clonogenic survival, and wound closure, and examined apoptosis- and cell cycle-related molecular markers by qRT-PCR and Western blotting. In addition, LC-MS/MS-based metabolomic profiling was performed to characterize compositional changes associated with selenium biofortification and to relate these changes to the observed biological activities.

It was hypothesized that Se biofortification would alter sunflower sprout composition and be associated with greater in vitro growth-inhibitory effects and modulation of apoptosis- and cell cycle-related markers in HT-29 cells compared with non-Se-enriched sprouts (control).

## 2. Materials and Methods

### 2.1. Production of Se-Enriched Sunflower Sprouts

The cultivation of Se-enriched sunflower sprouts was conducted according to the methodology described by Chantiratikul et al. [16]. *H. annuus* L. seeds were obtained from Rai Lung Top organic farm, Lopburi, Thailand (lot number 2023/12). Sprout production was performed in three independent biological batches per treatment. Initially, sunflower seeds were thoroughly cleaned and submerged in tap water for 9 h. Seeds were wrapped in damp woven cloth, fully covered, sealed inside airtight polyethylene bags, and incubated for 24 h. Afterwards, 125 g of germinated seeds were randomly cultivated in plastic trays (30 cm × 60 cm × 4 cm) filled with soil medium (Phuping brand, Buriram, Thailand). Se solutions were prepared from sodium selenate (Na_2_SeO_4_) at 0.0 mg/L (control sunflower sprout treatment) and 2.0 mg/L (Se-enriched sunflower sprout treatment). The Se solutions were applied at 500 mL per tray using a spray bottle. Next, the cultivated trays were covered with the identical inverted trays to prevent the germinated seeds from exposure to any light for 48 h.

Finally, the upper tray was removed, and the sunflower sprouts were exposed to sunlight and watered with 500 mL Se solutions daily at 08:00, 12:00 and 17:00. The average ambient temperature and relative humidity during cultivation were 23.3 ± 1.2 °C and 70 ± 9%, respectively. The sprouts were harvested by stem cutting at soil level on day 7 of cultivation and thoroughly washed with tap water. The Se concentrations in the control and Se-enriched sprouts were determined to be 48.21 ± 11.48 and 239.04 ± 4.02 mg/kg, respectively. Quantitative analysis was performed via a flame atomic absorption spectrometer (model 280FS) coupled with a VGA-77 hydride generation unit (Agilent Technologies, Inc., Santa Clara, CA, USA) at a wavelength of 196.0 nm. The analytical method exhibited a limit of detection (LOD) of 0.20 μg/L and a limit of quantification (LOQ) of 0.39 μg/L, with an average recovery of 98.45%.

### 2.2. Sunflower Sprout Extraction

The harvested sunflower sprouts were oven-dried at 40 °C until a constant dry weight was reached. The dried sprouts were ground to pass a 0.5 mm sieve. For extraction, dried sprout powder was mixed with 70% ethanol at a solid-to-solvent ratio of 1:10 (*w*/*v*) and subjected to maceration at 37 °C with shaking at 150 rpm for 24 h. The solvent was then removed using a rotary evaporator, and the crude extract was stored at −20 °C until further analysis. To improve batch-to-batch comparability, the sunflower sprout extracts were standardized based on dry extract concentration. The extraction yield from three biological replicates, calculated as the weight of dried crude extract relative to the weight of dried plant powder, was 19.65 ± 4.53% (*w*/*w*) for the control sunflower sprout extracts and 18.20 ± 5.41% (*w*/*w*) for the Se-enriched sunflower sprout extract.

### 2.3. Cytotoxicity Assay

The cytotoxic effects of the sunflower sprout extracts on HT-29 human colon cancer cells were evaluated using the MTT assay [18]. Cells were seeded at a density of 5000 cells/well in 96-well plates and incubated overnight. The dried sunflower sprout extracts were reconstituted in dimethyl sulfoxide (DMSO) and further diluted in culture medium such that the final DMSO concentration in all wells, including vehicle controls, did not exceed 0.1% *v*/*v*. Various concentrations of the sunflower sprout extracts (0, 200, 400, 600, and 800 µg/mL) in three biological replicates were applied for 24 h.

Following treatment, the medium was replaced with MTT solution and incubated for 4 h. Formazan crystals were dissolved with 200 µL of DMSO, and absorbance was measured at 590 nm. To assess possible colorimetric interference, extract-containing wells without cells were analyzed in parallel and their background absorbance was subtracted where applicable.

All reagents were obtained from Sigma-Aldrich (St. Louis, MO, USA). Cytotoxicity (%) was calculated using the formula:

$$\mathrm{Cytotoxicity}\;\left(\%\right)=\frac{\left(A_{o}-A_{e}\right)}{A_{o}}\times 100$$

where $A_{o}$ represents the absorbance of untreated cells and $A_{e}$ represents the absorbance of treated cells. Emax is the cytotoxicity (%). Half maximal inhibitory concentration (IC_50_) values were determined from in three biological replicates. Cytotoxicity toward normal human colon cells was assessed in the CCD 841 CoN cell line (ATCC^®^ CRL-1790™) using the MTT assay as described above. No positive control compound was tested in this assay.

The Selectivity Index (SI) is a measure of the selective cytotoxicity of a test compound toward cancer cells relative to normal cells. A higher SI value indicates greater selectivity for cancer cells and lower cytotoxicity toward normal cells, suggesting a more favorable safety profile. The SI was calculated using the following equation:

$$Selectivity\;Index\left(SI\right)=\frac{{IC}_{50}(Normal\;Cells)}{{IC}_{50}(Cancer\;Cells)}$$

### 2.4. Clonogenic Assay

The anti-colony formation activity of the sunflower sprout extracts was assessed as previously described [18]. HT-29 cells (500 cells/well) were seeded into six-well plates and allowed to attach for 24 h. The control or Se-enriched sunflower sprout extracts were then applied to the cells at doses of 0, 6.25, 12.5, 25, 50, 100, and 200 µg/mL in three biological replicates, followed by incubation at 37 °C in 5% CO_2_. After treatment, cells were washed twice with phosphate-buffered saline (PBS) and cultured in fresh DMEM for 14 days, with media replacement every 3 days. Colonies were fixed with methanol for 1 h and stained with 0.5% Coomassie Brilliant Blue G-250 for 30 min. Colonies were rinsed, photographed, and quantified. Colony formation percentage and IC_50_ values were calculated from triplicate experiments. No positive control compound was tested in this assay.

### 2.5. Wound-Healing Assay

To assess cell migration, a wound-healing assay was performed based on established protocols [19]. HT-29 cells were cultured in 24-well plates for a 24 h incubation period. A mechanical “wound” was created in the monolayer using a sterile 0.2 mL pipette tip. Cells were then assigned to untreated control groups, or treated with extract concentrations from 6.25 to 100 µg/mL. All treatments were run in three independent biological replicates. No positive control compound was tested in this assay.

Wound closure was documented via phase-contrast microscopy (NIB-9000, Xenon, Huizhou, China) at a 10× magnification over a 24 h window. The relative wound closure (%) was calculated as:

(1) $$Relative\;wound\;closure\left(\%\right)=\frac{\left(initial\;wound\;area-final\;wound\;area\right)}{\left(initial\;wound\;area\right)}\times 100$$

### 2.6. Gene Expression Analysis via Quantitative Real-Time Polymerase Chain Reaction (qRT-PCR)

HT-29 cells (2 × 10^5^ cells/well) were cultured overnight and treated with control or Se-enriched sunflower sprout extracts (50 µg/mL) for 24 h. The untreated cells were also included. Total RNA was isolated using TRIzol™ reagent (Thermo Fisher Scientific, Waltham, MA, USA), followed by cDNA synthesis. Gene expression levels were analyzed using a real-time PCR system (CFX96 Touch™, Bio-Rad, Watford, UK). Target gene expression was normalized to GAPDH and calculated using the 2^−ΔΔCT^ method. Each sample was analyzed in triplicate. The PCR conditions included an initial denaturation at 94 °C for 30 s, followed by 45 cycles of 94 °C for 15 s and 60 °C for 30 s using primers specific to the target genes (Table 1).

RNA purity and integrity were assessed by A260/A280 and gel electrophoresis. Primer efficiency for each gene was 90–100% and specificity was verified by melt-curve analysis. GAPDH was used as the reference gene after confirming stable expression under treatment conditions.

### 2.7. Protein Extraction and Western Blot Analysis

HT-29 cells were seeded at 2.5 × 10^5^ cells/well in 6-well plates and incubated for 24 h. Cells were treated with control or Se-enriched sprout extracts at 50 µg/mL for 24 h in three biological replicates. The untreated cells were also included. Cells were washed with ice-cold PBS and lysed in RIPA buffer containing protease and phosphatase inhibitors (Roche, Grenzach-Wyhlen, Germany) on ice for 30 min. Lysates were centrifuged, and protein concentration was determined using a BCA assay kit (Thermo Fisher Scientific, Waltham, MA, USA).

Protein samples (20 µg) were denatured at 98 °C for 10 min and separated by 12% SDS-PAGE, followed by transfer onto PVDF membranes at 90 V for 1 h. Membranes were blocked with 5% bovine serum albumin (BSA) in TBST for 1 h and subsequently incubated overnight at 4 °C with rabbit recombinant monoclonal anti-Bax antibody (1:1000, Cat. No. ab32503, Abcam, Cambridge, UK), rabbit recombinant monoclonal anti-p21 antibody (1:1000, Cat. No. ab109520, Abcam, Cambridge, UK), rabbit recombinant monoclonal anti-cyclin D1 antibody (1:1000, Cat. No. ab16663, Abcam, Cambridge, UK), and rabbit polyclonal anti-β-actin antibody (1:5000, Cat. No. ab8227, Abcam, Cambridge, UK). Following three washes with TBST, the membranes were incubated with an HRP-conjugated goat anti-rabbit IgG (H+L) secondary antibody (1:5000, Cat. No. 31460, Invitrogen, Thermo Fisher Scientific, Waltham, MA, USA) for 1 h at room temperature. Protein bands were detected using Amersham™ ECL Prime Western Blotting Detection Reagent (Cytiva, Marlborough, MA, USA) and visualized using a ChemiDoc Imaging System (Bio-Rad Laboratories, Hercules, CA, USA). Band intensities were quantified by densitometric analysis using Image Lab software version 6.1 (Bio-Rad Laboratories, Hercules, CA, USA), normalized to β-actin, and expressed relative to the untreated control. Data were obtained from three independent biological replicates.

### 2.8. Metabolomic Profiling via Liquid Chromatography–Tandem Mass Spectrometry (LC-MS/MS)

Sunflower sprout extracts (10 mg) were dissolved in 100% methanol containing 25 ng/mL sulfadimethoxine as an internal standard, reaching a final concentration of 25 mg/mL. To ensure data reliability, a quality control (QC) sample was generated by pooling 50 μL from each extract. This QC pool was further diluted to create a dilution series calibration set (0–100%). All prepared sunflower sprout extracts underwent centrifugation at 14,000 rpm for 10 min, and the supernatants were collected in LC vials. System stability and reproducibility were monitored by injecting the QC samples after every third sample injection [20].

Separation was achieved using an Agilent Poroshell 120 EC-C18 column (2.1 × 100 mm, 2.7 μm) at a stabilized temperature of 50 °C. The mobile phase, delivered at 0.4 mL/min, comprised 0.1% formic acid in water (Solvent A) and 0.1% formic acid in acetonitrile (Solvent B). A 10 μL sample volume was injected using a gradient program: initial 100% A (0–0.5 min), decreasing to 45% A (10.5 min), 25% A (12.5 min), and 0% A (14.0–17.0 min), before reverting to 100% A for a 2.5 min re-equilibration.

An Agilent 6545XT LC-QTOF system was employed for high-resolution detection in both ESI(+) and ESI(−) modes. The ESI source settings included a drying gas flow of 13 L/h at 325 °C and a sheath gas flow of 12 L/h at 275 °C, with a nebulizer pressure of 45 psi. Capillary voltages were set to 4000 V (positive) and 3000 V (negative). Scans were captured across 40–1700 *m*/*z* for MS1 and 25–1000 *m*/*z* for MS2. Collision energies were 20 eV for positive mode and 10 eV for negative mode.

Raw LC-MS/MS data were processed using MS-DIAL (v. 5.3). Peak detection and alignment were synchronized against a representative QC sample to standardize retention times and mass accuracy. LOWESS signal drift correction was performed prior to internal standard normalization. Metabolites were identified by comparing MS/MS fragments against the MS-DIAL ESI(±) libraries, the Fiehn/Vaniya Natural Product Library, and the BMDMS-NP database. Identification was confined to a 0.2–18 min retention window based on mass accuracy, molecular formulas, and spectral matches. For final analysis, data were filtered to include only features with a QC coefficient of variation < 30%, a sample-to-blank ratio ≥ 100, an identification score ≥ 0.70, and a Pearson correlation coefficient with the dilution series ≥0.70.

Log_2_ fold changes (FC) were calculated using normalized mean peak areas from Se-enriched and control sunflower sprout extracts, with each group analyzed in three biological replicates. A two-sample *t*-test was applied to identify metabolites with significant differences between treatments (*p* < 0.05). Significant differential metabolites were defined using thresholds of *p* < 0.05 and |log_2_ FC| ≥ 2. No multiple-comparison correction was applied in this exploratory study. Therefore, the reported *p*-values should be interpreted as nominal rather than adjusted.

Metabolite identities are reported as putative annotations based on spectral-library matching; positive-mode adducts included [M + H]^+^, [M]^+^, [M − H_2_O+H]^+^, and [M + Na]^+^, and the negative-mode adduct was [M − H]^−^. After the initial annotation step, all detected features underwent manual curation to ensure biological relevance. Features considered biologically irrelevant, misannotated, ambiguous, or non-endogenous were excluded from further analysis. To enhance annotation reliability, metabolite features with precursor mass errors exceeding 5 ppm were discarded prior to downstream analysis. A 5 ppm precursor mass error threshold was applied. This conservative threshold reduces ambiguous elemental composition matches, which is standard practice for untargeted plant metabolomics.

### 2.9. Statistical Analysis

Data are presented as the mean ± standard deviation (SD) from three independent biological replicates. Prior to parametric analysis, the assumptions of normality and homogeneity of variance were verified using the Shapiro–Wilk and Levene’s tests, respectively. Differences among treatment groups were analyzed using one-way analysis of variance (ANOVA), followed by Duncan’s multiple range test for post hoc comparisons. For concentration–response analyses, dose–response curves were fitted by nonlinear regression, and ${\mathrm{IC}}_{50}$ values were calculated using a four-parameter logistic model. Statistical computations were executed using SPSS software version 17.0 (IBM Corp., Armonk, NY, USA) and GraphPad Prism version 10.0 (GraphPad Software, Boston, MA, USA). A value of *p* < 0.05 was considered statistically significant.

## 3. Results

### 3.1. Cytotoxic Effects of Sunflower Sprout Extracts on HT-29 Cells

**Figure 1:**
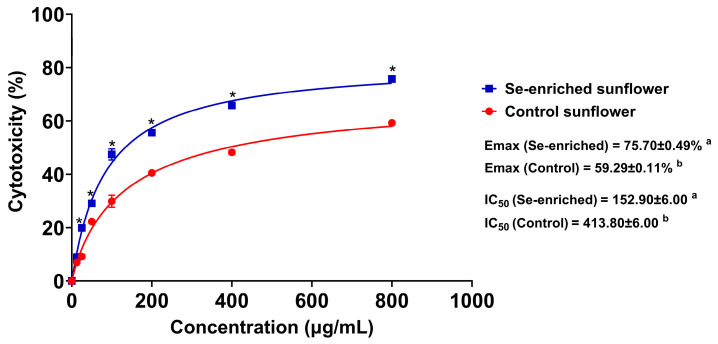
Cytotoxicity (%) of sunflower sprout extracts on HT-29 cells. The significant differences in cytotoxicity at each concentration of Se-enriched extract compared to control extract at *p* < 0.05 are labelled with an asterisk (*). Different superscripts for Emax and IC_50_ values indicate significant difference (*p* < 0.05).

The cytotoxic activity of control and Se-enriched sunflower sprout extracts against HT-29 human colorectal cancer cells was evaluated using the MTT assay (Figure 1). Both sunflower sprout extracts exhibited concentration-dependent cytotoxic effects; however, the Se-enriched extract exhibited significantly greater activity than the control extract. The control sunflower sprout extract showed an IC_50_ value of 413.80 ± 6.00 µg/mL, with a maximum cytotoxicity (Emax) of 59.29 ± 0.11%. Cytotoxicity gradually increased with concentration, reaching approximately 60% inhibition at 800 µg/mL. In contrast, the Se-enriched sunflower sprout extract exhibited a markedly lower IC_50_ value of 152.90 ± 6.00 µg/mL, indicating approximately 2.7-fold greater cytotoxic potency than the control extract. The Se-enriched extract also demonstrated a higher Emax value of 75.70 ± 0.49%, with cytotoxicity increasing rapidly at lower concentrations and reaching approximately 76% inhibition at 800 µg/mL. Overall, Se enrichment significantly enhanced the cytotoxic activity of sunflower sprout extracts against HT-29 colorectal cancer cells.

To assess biocompatibility and selectivity, the effects of the sunflower sprout extracts were evaluated in the human normal colon cell line CCD 841 CoN (ATCC CRL-1790™). Neither the control nor the Se-enriched sunflower sprout extract induced statistically significant cytotoxicity exceeding 50% across the tested concentration range in CCD 841 CoN cells (Supplementary Table S1 and Supplementary Figure S1).

IC_50_ values were >1500 µg/mL for both sunflower sprout extracts in normal colon cells. This yielded SI values of >3.6 for control and >6.5 for Se-enriched extracts. This indicated favorable selectivity toward HT-29 colorectal cancer cells, particularly for the Se-enriched sample. Samples with an SI value ≥ 3 are generally regarded as the minimum criterion for classifying a sample as a potential cancer cell growth inhibitor.

### 3.2. Antiproliferative Effects of Sunflower Sprout Extracts on HT-29 Cells

**Figure 2:**
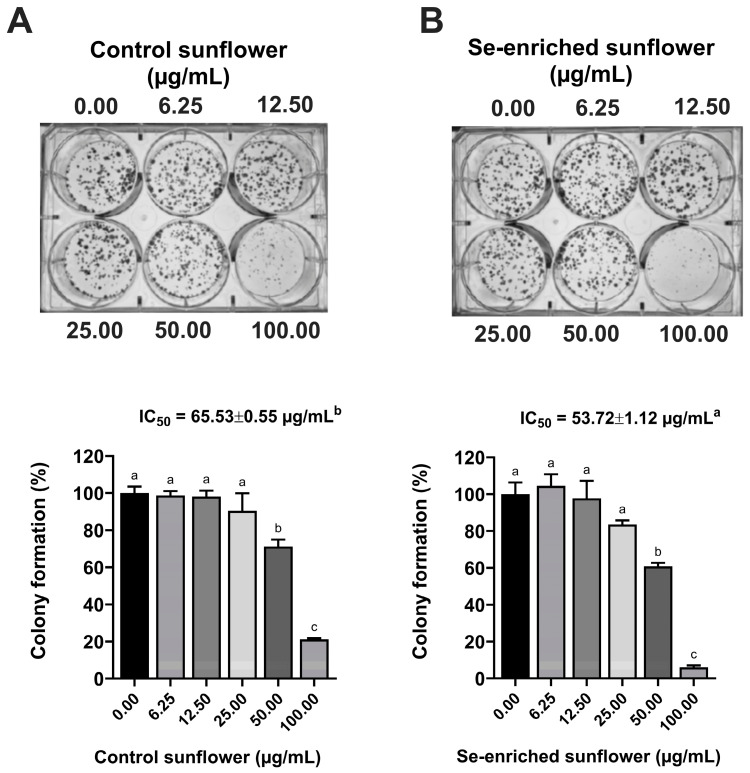
Colony formation (%) of HT-29 cells treated with sunflower sprout extracts. (**A**) Control extract. (**B**) Se-enriched extract. Different letters indicate significant difference between concentrations (*p* < 0.05). Different superscripts for IC_50_ values indicate significant difference (*p* < 0.05).

The effect of Se-enriched sunflower sprout extract on HT-29 colony formation was assessed and compared with the control (non-enriched) sprout extract (Figure 2).

Treatment with control sprout extract (Figure 2A) showed IC_50_ of 65.53 ± 0.55 µg/mL. In contrast, exposure to Se-enriched sunflower sprout extract (Figure 2B) led to a significantly lower IC_50_ of 53.72 ± 1.12 µg/mL (*p* < 0.05) suggesting a more pronounced inhibitory effect. The corresponding colony formation percentages confirmed the dose-dependent antiproliferative activity of the Se-enriched extract, whereas the control extract exhibited lower inhibition under the same conditions.

### 3.3. Inhibition of Wound Closure by Sunflower Sprout Extracts in HT-29 Cells

**Figure 3:**
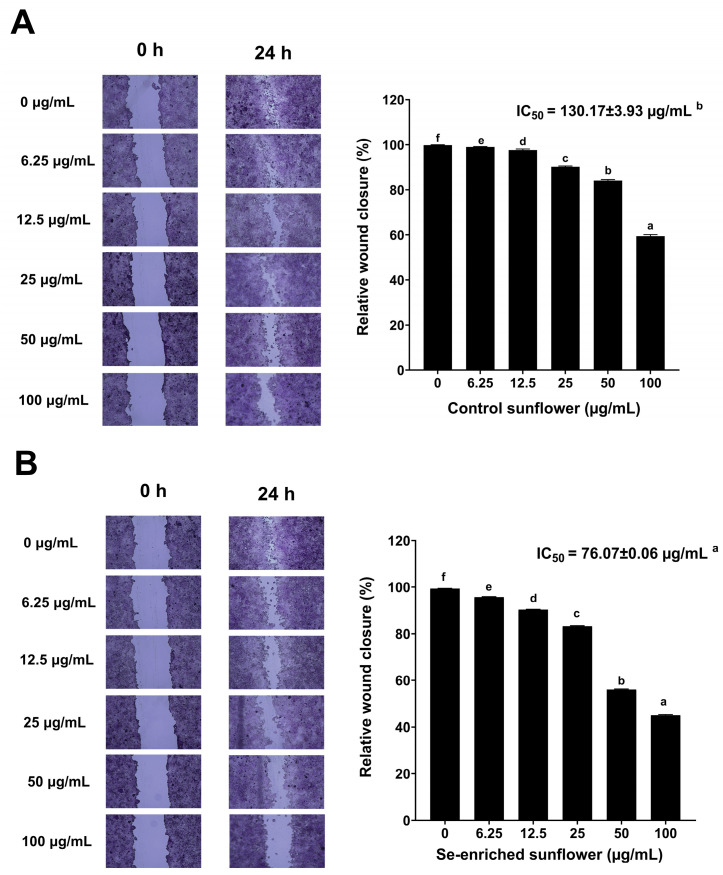
Relative wound closure (%) of HT-29 cells treated with sunflower sprout extracts. (**A**) Control extract. (**B**) Se-enriched extract. Different letters indicate significant difference between concentrations (*p* < 0.05). Different superscripts for IC_50_ values indicate significant difference (*p* < 0.05).

The wound closure capacity of HT-29 cells following treatment with sunflower sprout extracts was evaluated using a wound-healing assay (Figure 3).

Non-enriched control extract produced concentration-dependent wound closure reduction over 24 h. The calculated IC_50_ for the control extract was 130.17 ± 3.93 µg/mL (Figure 3A). In the Se-enriched group (Figure 3B), a more pronounced reduction in wound closure was observed compared to the control group at equivalent concentrations. The Se-enriched extract produced a concentration-dependent reduction in wound closure, with a significantly lower IC_50_ of 76.07 ± 0.06 µg/mL (*p* < 0.05).

These findings indicate that the Se-enriched extract was associated with greater inhibition of wound closure than the non-Se-enriched extract under the tested conditions. However, because mitomycin C was not used and the assay was not restricted to fully non-cytotoxic concentrations, the results should not be interpreted as definitive evidence of migration inhibition independent of cytotoxic effects.

### 3.4. Gene Expression in HT-29 Cells After Sunflower Sprout Extract Treatment

**Figure 4:**
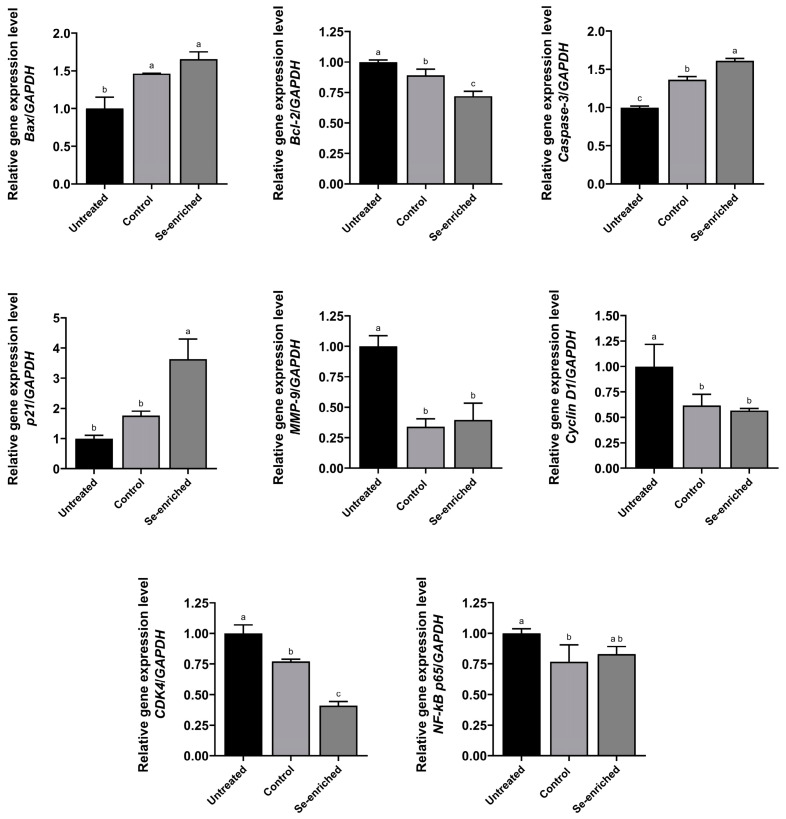
Relative gene expression levels in HT-29 cells treated with sunflower sprout extracts. Untreated = HT-29 cells without extract; Control = HT-29 cells with control extract; Se-enriched = HT-29 cells with Se-enriched extract. Different letters above the bars denote statistically significant differences among treatments (*p* < 0.05).

Treatment with Se-enriched sunflower sprout extract upregulated *Bax* and *caspase-3* transcripts and downregulated *Bcl-2*, consistent with apoptosis-related signaling (Figure 4). Concomitantly, *p21* was upregulated, and *CDK4* was downregulated, suggestive of G1 arrest, while *NF-κB p65* and *MMP-9* were downregulated, associated with suppression of pro-survival and pro-invasive pathways.

### 3.5. Protein Expression in HT-29 Cells After Sunflower Sprout Extract Treatment

**Figure 5:**
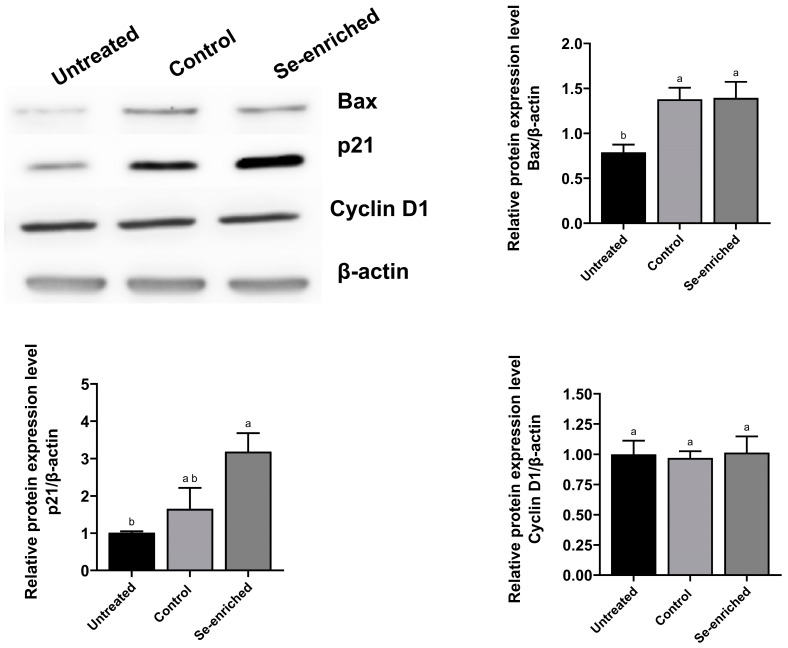
Relative protein expression levels in HT-29 cells treated with sunflower sprout extracts. Untreated = HT-29 cells without extract; Control = HT-29 cells with control extract; Se-enriched = HT-29 cells with Se-enriched extract. Different letters above the bars denote statistically significant differences among treatments (*p* < 0.05).

Western blotting was used to assess the protein expression levels of Bax, p21, and Cyclin D1 in HT-29 cells following treatment (Figure 5). Baseline expression of all three proteins was observed in untreated cells, while control extract treatment resulted in only moderate alterations. Notably, exposure to the Se-enriched extract markedly upregulated Bax and p21 expression compared to both the untreated and control groups. Conversely, Cyclin D1 protein levels remained unaffected across all groups. β-actin served as a loading control, confirming equal protein loading across all samples. These findings demonstrate that Se-enriched extract modulates the expression of key proteins, Bax and p21, associated with apoptosis-related signaling and cell cycle modulation, respectively, in HT-29 cells.

### 3.6. LC-MS/MS-Based Untargeted Metabolomic Profiling of Sunflower Sprout Extracts

**Figure 6:**
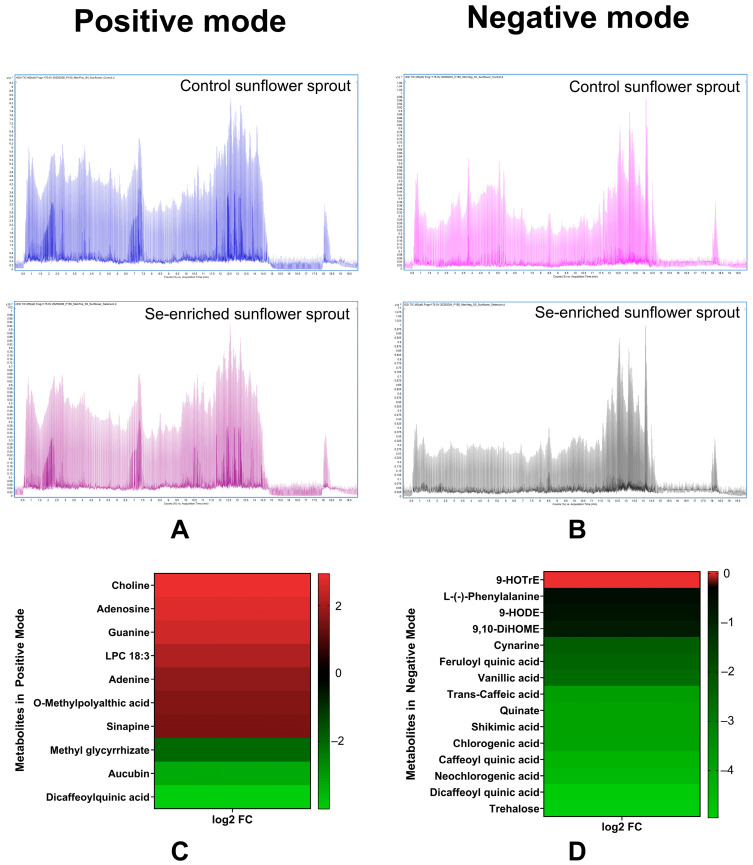
LC-MS/MS chromatograms and heatmaps of putative metabolites showing log_2_ FC in sunflower sprout extracts. (**A**) LC-MS/MS chromatograms of control and Se-enriched extracts in positive ion mode. (**B**) LC-MS/MS chromatograms of control and Se-enriched extracts in negative ion mode. (**C**) Heatmap of metabolites in positive ion mode. (**D**) Heatmap of metabolites in negative ion mode.

LC-MS/MS-based untargeted metabolite profiling identified 25 putatively annotated metabolites that satisfied the predefined quality-control and filtering criteria, including 10 metabolites detected in positive ion mode and 15 detected in negative ion mode (Figure 6, Table 2).

In positive ion mode, Se enrichment significantly increased the relative abundance of guanine (log_2_ FC = 2.54, *p* = 0.006), adenosine (log_2_ FC = 2.72, *p* = 0.003), lysophosphatidylcholine (LPC) 18:3 (log_2_ FC = 2.14, *p* = 0.012), and choline (log_2_ FC = 2.94, *p* = 0.001) compared with the control. In contrast, dicaffeoyl quinic acid (log_2_ FC = −3.99, *p* = 0.0004), aucubin (log_2_ FC = −3.29, *p* = 0.0009), and methyl glycyrrhizate (log_2_ FC = −2.01, *p* = 0.018) were significantly decreased. Other detected metabolites, including adenine, sinapine, and *O*-methylpolyalthic acid, did not differ significantly between treatments (*p* > 0.05).

In negative ion mode, Se enrichment was predominantly associated with reduced metabolite abundance. The most pronounced decreases were observed for trehalose (log_2_ FC = −4.96, *p* = 0.0001), dicaffeoylquinic acid (log_2_ FC = −4.91, *p* = 0.0001), neochlorogenic acid (log_2_ FC = −4.47, *p* = 0.0001), caffeoylquinic acid (log_2_ FC = −4.32, *p* = 0.0002), chlorogenic acid (log_2_ FC = −3.96, *p* = 0.0004), shikimic acid (log_2_ FC = −3.94, *p* = 0.0004), quinate (log_2_ FC = −3.92, *p* = 0.0004), trans-caffeic acid (log_2_ FC = −3.84, *p* = 0.0005), vanillic acid (log_2_ FC = −2.61, *p* = 0.005), feruloylquinic acid (log_2_ FC = −2.46, *p* = 0.007), and cynarine (log_2_ FC = −2.26, *p* = 0.010). No significant differences were detected for L-(−)-phenylalanine, 9-HOTrE, 9-HODE, or 9,10-DiHOME (*p* > 0.05).

Overall, Se enrichment was associated with increased levels of selected purine-related metabolites, LPC 18:3, and choline, together with decreased abundance of quinic acid derivatives and related phenolic metabolites. As this was an exploratory untargeted metabolomics study and no multiple-comparison correction was applied, the reported metabolites represent putative annotations and should be interpreted as hypothesis-generating findings pending confirmation by targeted metabolite identification and quantification.

## 4. Discussion

This study showed that Se biofortification enhanced the in vitro growth-inhibitory activity of sunflower sprout extracts against HT-29 colorectal cancer cells, relative to the non-Se control extract. The 2.7-fold reduction in IC_50_ and increase in maximal cytotoxicity indicate that Se enrichment substantially strengthens growth inhibition. However, the observed cytotoxic potency remains moderate and was not benchmarked against a standard chemotherapeutic or established positive control. Therefore, the findings should be interpreted as comparative proof-of-concept evidence rather than as validation of anticancer efficacy.

Similar Se-dependent gains in bioactivity have been reported in other biofortified foods and Se formulations, including Se-enriched sprouts and Se-based nanoparticles, which enhance cancer cell killing while sparing normal cells [12,15,21,22,23].

The colony-formation and wound closure results suggest that Se enrichment influenced HT-29 cell behavior beyond short-term viability measured by the MTT assay. However, the wound-healing assay was conducted over 24 h and without mitomycin C, so the reduced wound closure should not be interpreted as definitive evidence of an antimigratory effect independent of cytotoxicity or proliferation. Comparable anti-clonogenic effects, mediated by Se-dependent oxidative or mitochondrial stress, have been shown for Se nanoparticles and combined Se-based treatments in colorectal models [12,15,24,25].

Upregulation of *Bax* and *caspase-3* genes, along with downregulation of *Bcl-2* and an increased *Bax*/*Bcl-2* ratio, is associated with apoptosis-related signaling. This pattern has been reported for Se-containing systems and other anticancer agents across colorectal cancer models [12,21,22,23,25,26,27]. Concomitant induction of *p21* and suppression of *CDK4* and *Cyclin D1* further support enforcement of a G_1_ checkpoint and proliferative arrest, paralleling observations where natural products or Se-based nanomaterials increase *p21* and decrease *Cyclin* and *CDK* genes to halt the cell cycle [22,23,26,27]. The reduction of *NF-κB p65* and MMP-9 transcripts suggests additional targeting of pro-survival and pro-invasive pathways, in line with reports that Se formulations and plant extracts diminish NF-κB signaling and MMP activity to curb invasion and metastasis in colon cancer models [15,26,27,28]. However, direct functional assays, including Annexin V/PI staining, caspase activity assays, PARP cleavage, ROS measurement, mitochondrial membrane potential analysis, and flow-cytometric cell-cycle profiling, were not performed in this work and are needed to confirm the mechanism.

The LC-MS/MS-based metabolite profiling data provide a preliminary metabolic context for the observed bioactivities. In the Se-enriched sunflower sprouts, significant metabolic alterations were characterized by enriched levels of guanine, adenosine, LPC 18:3, and choline. Conversely, a marked depletion was observed for quinate, shikimic acid, and several quinic acid or phenolic derivatives, including dicaffeoylquinic acid, caffeoylquinic acid, neochlorogenic acid, chlorogenic acid, feruloylquinic acid, cynarine, vanillic acid, and *trans*-caffeic acid.

Recent metabolomic studies of Se biofortified legumes and microgreens also report that Se supplementation reshapes lipid and phenolic profiles, often elevating certain lipids while modulating phenolic pathways in a dose- and species-dependent manner [9,29,30,31,32]. In soybeans and mung beans, for example, Se biofortification alters phenylpropanoid-derived phenolics and linoleic-derived oxylipins, and can increase phenols at moderate doses while decreasing them at high exposure [9,29,32]. The strong downregulation of quinic/phenolic derivatives in Se-enriched sunflower sprouts suggests that Se may redirect carbon and reducing power away from these phenylpropanoid-related pools toward other metabolic sinks, such as purine salvage, membrane remodeling and Se amino acid synthesis [9,29,30,31,32]. Whether the observed upregulation of guanine, adenosine, LPC 18:3, and choline directly contributes to apoptosis-related signaling (e.g., via ATP/adenosine-dependent death pathways or membrane stress) remains a hypothesis, but the overlap with Se-induced metabolic reprogramming in other species supports a broader role of Se in tuning plant metabolomes and, consequently, bioactivity [9,29,30,31,32].

Although Se-enriched sunflower sprouts show a marked decrease in many phenolic acids, several mechanisms can explain their enhanced in vitro cancer cell growth-inhibitory potential.

Se biofortification drives incorporation of Se into organic forms such as selenoamino acids and Se polysaccharides, which often have higher anticancer activity than the original plant matrix. Selenylated polysaccharides or Se nanoparticles (SeNP)–polysaccharide conjugates show stronger growth inhibition and apoptosis in HT-29 than their non-Se counterparts, via *Bax/Bcl-2* shift, *caspase-3/8/9* activation, cytochrome c release, and ER stress/death receptor pathways [33,34]. Se-containing compounds in Se broccoli and other sprouts (e.g., MeSeCys) have been linked to enhanced apoptosis and reduced tumor growth in colon models, even when phenolic profiles are not increased [4,33,35].

Green synthesized or biogenic, SeNPs and Se polysaccharides induce strong ROS generation, mitochondrial membrane potential loss, sub-G1 arrest and 20–30% apoptosis in HT-29 at approximately 100 µg/mL, while sparing normal intestinal cells [14,33,34,36]. These effects are driven by Se-dependent redox modulation and selenoprotein-related pathways, not by phenolics [33,37,38]. Thus, even with fewer phenolic acids, Se-enriched sprouts can exert greater pro-oxidant and pro-apoptotic pressure specifically in cancer cells.

Se enrichment can reprogram metabolism, shifting from bulk phenolic acids to other bioactives (e.g., sulfur/Se compounds, specific phenolic subsets) without overall loss of chemopreventive potential [4,39,40]. In kale and kohlrabi sprouts, Se increased some phenolic acids while maintaining cytotoxicity at ≥1 mg/mL regardless of Se fortification [39]. Reviews on phenolic phytochemicals emphasize that specific structures, not total phenolic load, drive colorectal cancer inhibition via apoptosis and cell cycle arrest [41,42].

A reduction in total phenolic content does not preclude stronger in vitro cancer cell growth-inhibitory effects. In Se-enriched sunflower sprouts, enhanced activity is plausibly explained by formation of potent organ selenium species and Se-driven apoptotic signaling, plus qualitative shifts in phytochemicals, rather than phenolic quantity alone.

Although several phenolic and quinic acid derivatives decreased after Se biofortification, it was proposed that enhanced bioactivity may still arise from the emergence of Se-associated compounds or from qualitative shifts in other metabolite classes rather than from total phenolic abundance alone. Accordingly, reduced phenolic acid levels should not be interpreted as evidence of reduced functionality per se, but they do indicate that the mechanism of enhanced activity is unlikely to be explained simply by phenolic enrichment.

From a nutritional and functional foods perspective, these results align with a growing body of work, including Se-enriched sprouts and Se-rich microgreens, showing that Se biofortification can increase Se bioavailability and, at the same time, modify secondary metabolites without necessarily impairing antioxidant capacity or growth at appropriate doses [9,29,30,32,43,44]. Chantiratikul and co-workers have demonstrated that Se from plant-based biofortified sources, such as Se-enriched kale sprouts, is efficiently transferred into animal products and is often more bioavailable than inorganic sources, without adverse effects on performance [43,44,45]. The present work extends this concept by suggesting that Se-enriched sunflower sprouts may deliver not only improved Se nutrition but also enhanced in vitro cancer cell growth inhibition. This supports further evaluation of Se-enriched sunflower sprouts as a candidate functional food ingredient in preclinical models. Key translational gaps include Se speciation, extract standardization, gastrointestinal stability, bioaccessibility/bioavailability, realistic dietary intake estimates, dose-related safety, and in vivo efficacy.

However, these findings should be interpreted with caution since several limitations have been acknowledged. First, all experiments were conducted in vitro in HT-29 cells, which represent a single colorectal cancer model. Other colon cancer cell lines with different genetic backgrounds should be examined to confirm selectivity and generalizability, as shown in studies where Se nanoparticles were cytotoxic to colon cancer cells but spared normal intestinal cells [15,23]. Additionally, the cellular assays did not include a positive control compound, which limits contextualization of effect size. Second, although the transcriptional and protein data implicate Bax/Bcl-2, caspases, p21/CDK4/Cyclin D1 and NF-κB/MMP-9, direct measurements of caspase activity, mitochondrial membrane potential, and ROS formation would strengthen the causal link to intrinsic apoptosis, as reported for Se-containing nanomaterials and Se co-treatments in colon cancer models [13,15,21,22,23,24,25]. Third, the metabolomic analysis was restricted to a focused set of 25 metabolites; wider untargeted profiling and pathway analysis could better connect Se-induced metabolic remodeling to the observed phenotypes, building on comprehensive metabolomic work in Se-enriched legumes and microgreens [9,29,30,31,32]. Fourth, in vivo studies in appropriate colorectal tumor models are needed to determine whether the doses of Se and sprouts used here translate into effective and safe chemopreventive or adjuvant regimens. Finally, Se chemical speciation was not performed; therefore, the specific chemical forms responsible for the observed bioactivities remain unknown. Furthermore, because the metabolite profiling was an exploratory untargeted analysis without multiple-comparison correction, these identities should be interpreted as putative annotations requiring further validation. Accordingly, any link between these metabolic shifts and the observed cellular effects remains suggestive rather than mechanistically confirmed.

The sunflower sprout extracts showed greater cytotoxic activity toward HT-29 colorectal cancer cells than toward the normal colon cell model under the tested in vitro conditions. This is consistent with prior work on Se-enriched extracts and Se nanoparticles, which demonstrated preferential cytotoxicity towards cancer cells with negligible activity against normal cells [46,47,48]. These findings suggest a potentially favorable in vitro selectivity profile for sunflower sprout extracts, although this should be confirmed in additional cancer and normal cell models. All findings in this study were obtained in vitro using HT-29 cells and should not be interpreted as direct evidence for colorectal cancer prevention in humans. In vivo studies and bioavailability assessment are required before any preventive or translational claims can be made.

## 5. Conclusions

Se biofortification enhanced the in vitro biological activity of sunflower sprout extracts in HT-29 cells. In line with the study objectives, the Se-enriched extract showed greater cytotoxicity, lower clonogenic survival, and reduced wound closure compared to the non-enriched extract (control), with IC_50_ values of approximately 152.90 µg/mL for cytotoxicity, 53.72 µg/mL for colony formation, and 76.07 µg/mL for wound closure inhibition. These effects were accompanied by changes in apoptosis- and cell cycle-related markers, including modulation of *Bax/Bcl-2*, *caspase-3*, *p21*, *CDK4*, *NF-κB p65*, *MMP-9*, and *Cyclin D1*, as well as a distinct LC-MS/MS-based metabolite profile. Se biofortification also altered sprout composition, with higher levels of selected purine-related metabolites, lysophospholipids, and choline, alongside lower levels of several phenolic and quinic acid derivatives. However, these molecular changes are only consistent with apoptosis- and growth-related pathway involvement in this cell model and do not by themselves confirm mitochondrial apoptosis or specific cell-cycle arrest mechanisms. Furthermore, because the metabolite profiling was an exploratory untargeted analysis without multiple-comparison correction, these metabolite identities should be interpreted as putative annotations requiring further validation. Accordingly, any link between these metabolic shifts and the observed cellular effects remains suggestive rather than mechanistically confirmed. These findings should be interpreted in light of several limitations. The study used only a single in vitro cell model, Se speciation was not determined, and direct functional assays for apoptosis and cell-cycle progression were not performed. In addition, no in vivo validation was included. Therefore, the results do not support direct conclusions regarding colorectal cancer prevention or efficacy in humans. Overall, the findings provide proof-of-concept that Se biofortification can modify sunflower sprout composition and enhance growth-inhibitory effects in HT-29 cells under in vitro conditions. Future studies should identify Se species, validate apoptosis- and cell cycle-related mechanisms using functional assays, assess bioavailability and safety, and confirm these effects in animal models and additional colorectal cancer cell systems.

## Figures and Tables

**Table 1:** Primer sequences used for gene expression analysis via qRT-PCR.

Gene	Role	Primer	Sequence (5′-3′)	Size (bp)
*GAPDH*	Housekeeping gene	Forward	GGATTTGGTCGTATTGGGCG	115
		Reverse	TCCCGTTCTCAGCCATGTAG	
*Bax*	Pro-apoptotic	Forward	GAGCAGCCCAGAGGCG	276
		Reverse	AGCTGCCACTCGGAAAAAGA	
*Bcl-2*	Anti-apoptotic	Forward	ATGTGTGTGGAGAGCGTCAA	135
		Reverse	ATCACCAAGTGCACCTACCC	
*Caspase-3*	Caspase pathway	Forward	GTGCTATTGTGAGGCGGTTG	271
		Reverse	GTTTCCCTGAGGTTTGCTGC	
*p21*	Cell cycle inhibition	Forward	CCCAACGCACCGAATAGTTAC	167
		Reverse	GAAAACTCCCCAGGAAGCCT	
*MMP-9*	Tumor invasion and metastasis	Forward	TATGACATCCTGCAGTGCCC	111
		Reverse	TTGTATCCGGCAAACTGGCT	
*Cyclin D1*	G_1_/S phase transition in the cell cycle	Forward	GCTGTAGTGGGGTTCTAGGC	297
		Reverse	AGCGTATCGTAGGAGTGGGA	
*CDK4*	Partners with Cyclin D1 to drive cell cycle progression	Forward	GTATGGGGCCGTAGGAACC	113
		Reverse	AGGCAGAGATTCGCTTGTGT	
*NF-κB p65*	Regulating inflammation and survival	Forward	CTGCACTGTGGGGTCACAT	114
		Reverse	GGACACTTGAATCAGCAGGC	

Eight genes were analyzed, while protein validation focused on only 3 proteins: Bax, p21, and Cyclin D1.

**Table 2:** Putative metabolite annotations in sunflower sprout extracts (Se-enriched and control) via LC-MS/MS-based untargeted profiling in both positive and negative modes.

Met ID	RT (min)	*m*/*z*	Metabolite Name	Adduct	Ontology	Score	S/N Average	Blank	Control Extract	Se-Enriched Extract	Mass Error (ppm)	Log_2_ FC	*p*-Value
Positive mode													
1301	2.351	136.06351	Adenine	[M + H]^+^	6-aminopurines	1.65	258.3	8836.17	1,139,300.95	3,870,912.64	2.57	1.76	0.084
13121	11.813	518.32324	Lysophosphatidylcholine (LPC) 18:3	[M + H]^+^	1-acyl-sn-glycero-3-phosphocholines	1.63	502.83	232.94	200,118.78	884,909.07	1.76	2.14	0.012
1718	2.395	152.05693	Guanine	[M + H]^+^	Purines and purine derivatives	1.56	88.52	2053.62	28,548.71	165,507.01	−0.53	2.54	0.006
5136	2.351	268.10388	Adenosine	[M + H]^+^	Purine nucleosides	1.47	133.51	36.32	233,619.96	1,536,594.19	−0.45	2.72	0.003
6546	3.349	310.16489	Sinapine	[M]^+^	Coumaric acids and derivatives	1.45	1074.19	0	44,652.47	127,652.38	−1.77	1.52	0.125
12607	5.845	499.12329	Dicaffeoyl quinic acid	[M − H_2_O+H]^+^	Quinic acids and derivatives	1.39	85.84	0	2,852,595.98	179,468.95	0.6	−3.99	0.0004
520	0.631	104.10661	Choline	[M]^+^	Cholines	1.30	667.11	13,572.49	1,805,330.13	13,836,968.08	−3.64	2.94	0.001
7859	3.818	347.13132	Aucubin	[M + H]^+^	Iridoid O-glycosides	1.09	85.89	0	3,275,008.82	335,779.67	−4.86	−3.29	0.0009
19449	11.114	861.40784	Methyl glycyrrhizate	[M + Na]^+^	Triterpene saponins	1.05	965.9	0	2,393,723.91	594,142.44	−1.77	−2.01	0.018
8025	9.951	351.25293	O-Methylpolyalthic acid	[M + H]^+^	Diterpenoids	1.05	46.94	0	1,494,343.33	4,577,637.51	−2.16	1.62	0.091
Negative mode													
1307	2.811	164.07153	L-(-)-Phenylalanine	[M − H]^−^	Phenylalanine and derivatives	1.63	396.05	19.27	7,477,288.55	5,250,835.02	2.19	−0.51	0.420
5243	3.801	353.09079	Caffeoyl quinic acid	[M − H]^−^	Quinic acids and derivatives	1.57	62.11	10.64	14,434,310.8	721,258.88	2.97	−4.32	0.0002
3867	11.991	293.22687	9-HOTrE	[M − H]^−^	Lineolic acids and derivatives	1.57	2089.42	0	11,347,025.15	11,699,631.50	4.07	0.04	0.915
3902	13.37	295.22916	9-HODE	[M − H]^−^	Lineolic acids and derivatives	1.53	1998.17	381.98	15,694,909.55	10,011,048.91	4.23	−0.65	0.310
4937	0.706	341.1084	Trehalose	[M − H]^−^	O-glycosyl compounds	1.51	102.43	105.12	4,064,679.28	130,801.15	−1.44	−4.96	0.0001
1553	5.848	179.03461	*trans*-Caffeic acid	[M − H]^−^	Hydroxycinnamic acids	1.51	42.36	10.15	2,511,717.99	175,666.78	−2.12	−3.84	0.0005
9395	5.213	515.12177	Cynarine	[M − H]^−^	Quinic acids and derivatives	1.49	67.33	0	11,743,386.97	2,456,263.18	4.39	−2.26	0.010
5238	3.244	353.08777	Neochlorogenic acid	[M − H]^−^	Quinic acids and derivatives	1.48	50.57	0	11,946,243.51	537,912.33	−0.08	−4.47	0.0001
5242	5.588	353.09027	Chlorogenic acid	[M − H]^−^	Quinic acids and derivatives	1.45	73.18	0	11,801,700.39	760,569.57	2.71	−3.96	0.0004
1774	5.843	191.05576	Quinate	[M − H]^−^	Quinic acids and derivatives	1.44	46.17	37.96	2,075,220.85	137,201.01	−1.26	−3.92	0.0004
1459	5.846	173.04512	Shikimic acid	[M − H]^−^	Shikimic acids and derivatives	1.41	94.48	0	2,827,776.05	183,714.74	−2.2	−3.94	0.0004
5573	3.989	367.10358	Feruloyl quinic acid	[M − H]^−^	Quinic acids and derivatives	1.13	330.72	0	125,008.90	22,797.88	0.52	−2.46	0.007
9393	4.669	515.11981	(1S,3R,4S,5R)-3,5-bis({[(2E)-3-(3,4-dihydroxyphenyl)prop-2-enoyl]oxy})-1,4-dihydroxycyclohexane-1-carboxylic acid (Dicaffeoyl quinic acid)	[M − H]^−^	Quinic acids and derivatives	1.08	20,870.82	0	7,971,482.45	264,678.98	1.77	−4.91	0.0001
1361	2.998	167.03461	Vanillic acid	[M − H]^−^	M-methoxybenzoic acids and derivatives	1.08	30.54	20.56	1,293,864.83	212,380.84	−4.07	−2.61	0.005
4321	13.275	313.23825	9,10-DiHOME	[M − H]^−^	Long-chain fatty acids	1.01	130.2	6.01	2,123,199.98	1,204,962.21	−0.57	−0.82	0.185

Abbreviations: log_2_ FC = log_2_ Fold Change; S/N = Signal-to-noise ratio. Putative metabolite annotation was restricted to a 0.2–18 min retention window using mass accuracy, molecular formula, and spectral matching. Features retained for analysis met the following criteria: QC coefficient of variation less than 30%, sample-to-blank ratio ≥ 100, identification score ≥ 0.70, Pearson correlation with the dilution series ≥ 0.70, and mass error ≤ 5 ppm. The ${log}_{2}$ FC was calculated from normalized mean peak areas of Se-enriched vs. control sunflower sprouts (*n* = 3 biological replicates per group). Significant metabolites were defined by p<0.05 and fold change ≥ 2. No multiple-comparison correction was applied for this exploratory, untargeted analysis; reported p-values are nominal. These metabolite identities should be interpreted as putative annotations requiring further validation. All annotated features were manually curated; biologically irrelevant, misannotated, ambiguous, or non-endogenous features were excluded.

## Data Availability

The original contributions presented in the study are included in the article/Supplementary Materials; further inquiries can be directed to the corresponding author.

## Supplementary Materials

The following supporting information can be downloaded at: https://www.mdpi.com/article/10.3390/foods15142539/s1, Table S1: Cytotoxicity of sunflower sprout extracts on human normal colon cells (CCD-841 CoN); Figure S1: Cytotoxicity of sunflower sprout extracts on human normal colon cell line CCD 841 CoN (CRL-1790 ™ from ATCC). (A) Control sunflower sprout treatment. (B) Se-enriched sunflower sprout treatment. Both treatments show IC50 values of >1500 µg/mL.
