# Supplementary material for: From Biofortification to HT-29 Growth Inhibition: Selenium-Enriched Sunflower Sprouts Modulate Apoptosis- and Cell Cycle-Related Markers
Source: Foods. 2026 Jul 17;15(14):2539. doi: 10.3390/foods15142539 (PMC13408742; doi:10.3390/foods15142539)
Supplement: Supplementary file 1 [file foods-15-02539-s001.zip › foods-4390665-supplementary.pdf]

Table S1. Cytotoxicity of sunflower sprout extracts on human normal colon cells (CCD-841 CoN).

Concentration (µg/mL)	% Cytotoxicity (Mean ± SD) of Control Sunflower Extract	% Cytotoxicity (Mean ± SD) of Se-Enriched Sunflower Extract
0	0.00 ± 0.00 [a]	0.00 ± 0.00 [a]
12.5	1.25 ± 0.50 [a]	2.10 ± 0.85 [a]
25	3.40 ± 1.20 [a]	4.85 ± 1.10 [a]
50	5.80 ± 0.95 [b]	8.30 ± 1.45 [a]
100	9.15 ± 1.60 [b]	13.50 ± 2.10 [a]
200	14.20 ± 2.35 [b]	21.40 ± 1.80 [a]
400	20.80 ± 1.90 [b]	26.65 ± 2.40 [a]
800	28.50 ± 2.70 [b]	34.10 ± 3.15 [a]

*Superscripts in the rows indicate statistical differences ($p < 0.05$).

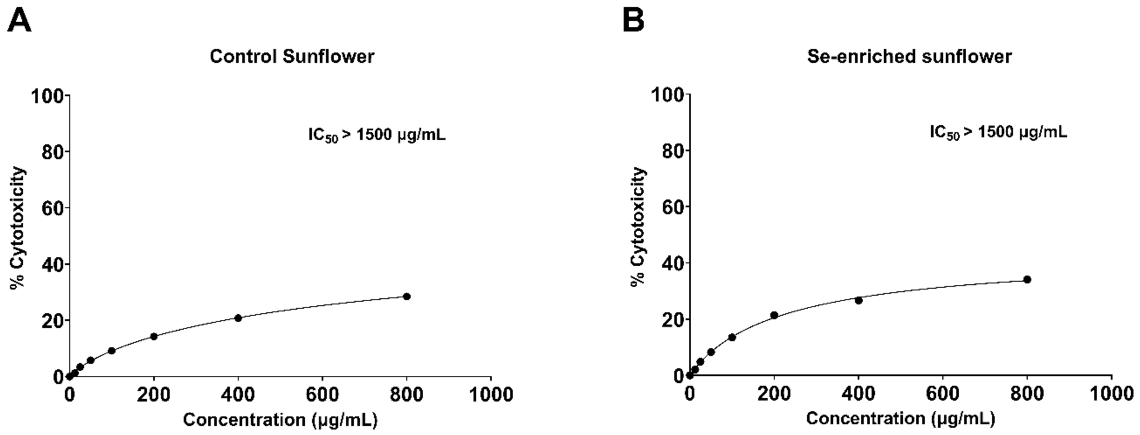

Figure S1. Cytotoxicity of sunflower sprout extracts on human normal colon cell line CCD 841 CoN (CRL-1790 ™ from ATCC). (A) Control sunflower sprout treatment. (B) Se-enriched sunflower sprout treatment. Both treatments show IC_{50} values of > 1500 µg/mL.
